# Fitting Biochars and Activated Carbons from Residues of the Olive Oil Industry as Supports of Fe- Catalysts for the Heterogeneous Fenton-Like Treatment of Simulated Olive Mill Wastewater

**DOI:** 10.3390/nano10050876

**Published:** 2020-05-01

**Authors:** Bruno M. Esteves, Sergio Morales-Torres, Francisco J. Maldonado-Hódar, Luis M. Madeira

**Affiliations:** 1 LEPABE—Laboratory for Process Engineering, Environment, Biotechnology and Energy, Faculty of Engineering, University of Porto, Rua Dr. Roberto Frias, 4200-465 Porto, Portugal; ega11010@fe.up.pt; 2 Department of Inorganic Chemistry, Faculty of Sciences, University of Granada, Avda. Fuente Nueva, 18071 Granada, Spain; semoto@ugr.es

**Keywords:** agricultural residues, biochars, activated carbons, Fe-carbon catalysts, CWPO, olive mill wastewater, Fenton

## Abstract

A series of biochars and activated carbons (ACs) was prepared combining carbonization and physical or chemical activation of cheap and abundant residues of the olive oil industry. These materials were used as Fe-support to develop low-cost catalysts for the heterogeneous Fenton-like oxidation of simulated olive mill wastewater (OMW), the highly pollutant effluent generated by this agroindustry. Commercial ACs were also used as reference. All catalysts prepared were extensively characterized and results related with their performances in the catalytic wet peroxide oxidation (CWPO). Results showed a linear relationship of the textural properties of the catalysts with the adsorptive and catalytic performance, as well as the preferential adsorption and degradation of some phenolic compounds (caffeic and gallic acids) by specific interactions with the catalysts’ surface. Despite the best performance of catalysts developed using commercial supports, those prepared from agro-industrial residues present some advantages, including a smaller catalyst deactivation by iron leaching. CWPO results show that catalysts from physically activated olive stones are the most promising materials, reaching total organic carbon and toxicity reductions of 35% and 60%, respectively, as well an efficient use of H_2_O_2_, comparable with those obtained using commercial supports. This approach showed that the optimized treatment of this type of residues will allow their integration in the circular economic process of the olive oil production.

## 1. Introduction

Pollution of water bodies and scarcity of clean water is an increasing worldwide issue related to population growth, excessive urbanization and industrialization, as well as climate changes. Industrial wastewaters are among the main polluting agents of water sources, being the food processing industry one of the highest potable-water consuming activity per ton of product processed [1]. According to the Food and Agriculture Organization Corporate Statistical Database (FAOSTAT) on crops around the globe, the water footprint of olive oil’s processing industry can reach 14,500 m^3^ per ton of oil extracted [2]. Depending on the olive oil extraction process employed, the amount of wastewater generated may be as high as 50% of the total water inputs.

The olive oil sector is particularly important for countries in the Mediterranean region, where ~3.3 million metric tons of olive oil were extracted in the 2018 campaign, according to the International Olive Oil Council (IOOC) [3]. The volume and intrinsic characteristics of olive mill wastewaters (OMW) strongly varies according to the extraction process employed, olive variety, climate conditions, cultivation practices, and storage. Nonetheless, OMW is commonly known for its slight acidic pH, dark-red to brown color and foul smell, being mostly comprised of water (83–94% *w/w*) and organic matter (4–16% *w/w*), including polyphenols, organic acids, lipids, tannins and reduced sugars. Phenolic compounds of olive’s stones and pulp are particularly important thanks to their greater solubility in water than oil, leading to the high concentration (2–15% *w/w*) observed in such effluents [4,5]. It has been reported that phenolic compounds, along with some organic acids such as the acetic and formic acids, are responsible for OMW’s phytotoxic and antibacterial effect [6,7]. 

Due to the high organic load, turbidity and biorecalcitrant nature of many organic compounds present in OMW, proper management of the effluents generated is techno-economically challenging for most small- and medium-sized mills, which compose the geographically dispersed olive oil industrial sector. Although composting or valorization for energy purposes are possible, most OMW ends up in evaporation ponds or dispersed in agricultural lands due to the high volume of effluents generated, seasonality of production and lack of central management among olive mill facilities [8]. Improper storage or uncontrolled discharge of OMW may pose serious environmental threats related to proliferation of odor nuisance and soil and aquifers contamination, resulting in plants growth inhibition and overall imbalance of aquatic ecosystems (e.g., discoloration of water bodies, eutrophication, and/or oxygen depletion) [9,10].

Several treatment processes have been proposed to address this issue, including physicochemical procedures such as coagulation/flocculation [11], adsorption [12] or electrocoagulation [13], aerobic and anaerobic biological processes [14,15,16], as well as a wide range of advanced oxidation processes (AOPs), namely ozonation [17], Fenton-based [18], photocatalytic [19] or electrochemical [20] processes. However, the applicability and efficiency of AOP single-step units is often limited by OMW characteristics, as influent values of chemical and biochemical oxygen demand (COD, BOD_5_) and total suspended solids (TSS) may be as high as 200, 100, and 35 g/L, respectively [21]. Likewise, bio-remediation processes may be hindered by the presence of biorefractory and recalcitrant compounds such as phenolic acids. Evidently, the adoption of integrated treatment schemes is often suggested as the most cost-effective alternative to meet discharge criteria regulations. In that sense, many proposed schemes include a pre-treatment unit to reduce the initial organic load, for instance acid cracking, oil separation and coagulation/flocculation units [22,23,24], while AOPs are often suggested to reduce the toxic character of OMW prior to biological treatment [25,26,27].

AOPs are well-known technologies capable of mineralizing a wide range of pollutants thanks to the high reactivity and non-selectivity of the generated hydroxyl radicals (^•^OH). One of the most cost-efficient AOPs is the Fenton process, which relies on the ability of Fe^2+^ ions to act as catalyst for H_2_O_2_ decomposition in ^•^OH at near-ambient temperature and atmospheric pressure [28]. The major drawbacks associated with the classic (homogeneous) Fenton process are the limited pH range of operation (usually from 2–4) and the formation of an iron-rich sludge that needs to be handled downstream. Naturally, attention from researchers has shifted towards the “immobilization” of iron (as well as other transition metals) within a solid-matrix (support) as to address such constrains. The major goal in this heterogeneous Fenton-like process—also known as catalytic wet peroxide oxidation (CWPO)—has been to prepare active and stable catalysts capable of inducing the decomposition of H_2_O_2_ to ^•^OH, as well as to modify the catalysts’ physicochemical properties to match specific treatment requirements [29].

Several supports, including clays [30,31], mesoporous silica [32,33], zeolites [34,35], and activated carbons (ACs) [36,37,38] have been used to develop Fe-based catalysts (as well as other such as Cu, Cr, or Mn) for the heterogeneous Fenton-like reaction of different target compounds and wastewaters. Carbon materials, and particularly ACs, are widely known for their versatility in environmental applications, namely in water and wastewater depuration as adsorbents of contaminants [39,40], catalysts on their own [41], and/or catalyst’s supports [36,42,43]. The well-developed porous structure and high surface area of activated carbons, along with the presence of oxygen surface groups responsible for the metallic phase’s anchorage and dispersion, are some of the advantages of such materials [44].

The application of CWPO for olive mill wastewater/phenolic wastewaters treatment has been reported in the literature. Martins et al. [45] prepared different ceria-based catalysts (Fe-Ce-O) for the depuration of a mixture comprising different phenolic compounds, achieving 57% mineralization after 120 min., using 1 g/L of catalyst and 7.6 g/L of H_2_O_2_. Furthermore, the same authors [46] tested this catalyst on weathered OMW oxidation—total organic carbon (TOC) = 309 mg/L, COD = 1700 mg/L—achieving 25% COD removal under optimized operational conditions (pH = 4, [Cat.] = 1.5 g/L and [H_2_O_2_] = 3.9 g/L). Najjar et al. [47] have also tested a series of beta-zeolites for the catalytic oxidation of a diluted OMW (TOC_0_ = 1300 mg/L), achieving 28% TOC and 40% total phenols (TPh) reductions after 12 h, with 30% decrease in the effluent’s toxicity towards *Vibrio fischeri* bacteria. More recently, Maduna et al. [48] have reported on OMW’s phenols oxidation kinetics over a series of copper-zeolite catalysts.

Solid by-products such as olive stones and olive tree pruning, as well as semi-solid residues usually known as pomaces, also require management alternatives, as they comprise an important fraction of the total residues generated by olive mills. Some authors have already tested such residues as precursors for the preparation of adsorbents, with applications on adsorption of OMW contaminants [39,49] or heavy metals removals from water [50,51]. In this work, the catalytic activity of different Fe-based catalysts, prepared from olive stones and olive tree pruning’s sawdust biochars, is evaluated for the treatment of a phenolic mixture simulating the polyphenolic composition of real OMW. The activity and stability of the synthetized materials will be compared to commercially available ones. Performance of the CWPO process will be monitored following the solution’s mineralization degree (total organic carbon—TOC—removal), degradation of individual parent phenolic compounds and H_2_O_2_ consumption efficiency. The bioluminescence inhibition of *V. fischeri* bacteria will be used as a toxicity indicator, and the Fe leaching assessed to infer on the catalysts stability. The goal of this work is to check the suitability of two olive oil extraction by-products as Fe-support catalysts on the oxidation of OMW’s characteristic phenolic compounds and reduction of the solution’s overall toxicity, in the perspective of circular and sustainable economy, which to the best of author’s knowledge has never been reported yet.

## 2. Materials and Methods

### 2.1. Catalysts Synthesis

Catalysts were prepared using two agricultural by-products from the olive oil extraction operation: olive stones (OS) and sawdust (SD) from olive tree pruning. The raw starting materials were provided by local manufacturers from the region of Granada, Spain. Prior to use, olive stones and sawdust were thoroughly washed, ground and sieved to a particle size fraction of 0.45–1.0 mm.

Biochars were prepared through the carbonization of the raw materials using a horizontal tube furnace at 800 °C (heat ramp of 10 °C/min, 2 h of hold time) under N_2_ flow (flow rate of 150 cm^3^/min). All volumetric flow rates were measured at room temperature and atmospheric pressure. Physically activated biochars were obtained using CO_2_ as the activating agent. For that, the flow of N_2_ was exchanged for CO_2_ (flow rate of 300 cm^3^/min and 4 h of hold time) after the carbonization period, and finally the oven turned-off and the sample allowed to cool to room temperature, again under an inert (N_2_) atmosphere. These samples were denominated indicating the thermal treatments of the corresponding raw materials, thus OSC and SDC correspond to biochar from OS and SD, while OSC-AC and SDC-AC to the physically activated biochars, respectively. Additionally, another fraction of OS was chemically activated, using KOH as activating agent (sample OS-AC/KOH). For that, a mixture of OS/KOH was prepared in a weight ratio of 1 and thermally treated at 800 °C for 2 h and allowed to cool to room temperature. Samples were finally washed with diluted HCl and then with distilled water until absence of chloride ions in the washing waters was observed.

Fe-supported catalysts were then prepared by incipient wetness impregnation (IWI) using the appropriate amount of FeCl_2_∙4H_2_O (Acros Organics, Geel, Belgium) aqueous solution as the metal precursor, to obtain an iron load of 5 wt.%. Impregnated samples were dried overnight at 100 °C, then treated for 1 h under N_2_ flow of 150 cm^3^/min at 350 °C (heat ramp of 10 °C/min), and finally stored in air-tight sealed containers until needed. For comparative purposes, two commercial activated carbons were also used as Fe-supports: one from Norit (Norit RX 3 Extra; Sigma Aldrich, St. Louis, MO, USA) and the other from Merck (ref. 102,514 AC pure; Darmstadt, Germany), commercialized in granular form, which were also grounded and sieved to the particle size fraction of 0.45–1.0 mm. The designation and synthesis conditions of each catalyst used in this study are reported on Table 1.

### 2.2. Catalysts Characterization

Surface area and pore size analysis were performed by physisorption of N_2_ and CO_2_ at 77 K and 273 K, respectively (Quantachrome Quadrasorb *SI*, Boynton Beach, FL, USA), with samples previously degasified overnight at 120 °C. The apparent surface area (*S_BET_*) was calculated applying the BET equation [52] to N_2_ adsorption isotherms (*P/P*_0_ < 0.10). The micropore volume (*W*_0_) and the mean micropore width (*L*_0_) were obtained by the Dubinin-Radushkevich and Stoeckli equations, respectively [53]. The total pore volume (*V_T_*) was obtained considering the volume of N_2_ adsorbed at *P/P*_0_ = 0.95, whilst the mesopore volume (*V_meso_*) from the difference between *V_T_* and the volume adsorbed at *P/P*_0_ = 0.40 by application of the Gurvich rule [54]. Quenched Solid Density Functional Theory (QSDFT) method, assuming slit-shaped pores, was applied to N_2_ isotherms to derive the pore size distribution (PSD) of samples [55].

The materials’ morphology was studied by high-resolution scanning electron microscopy (HRSEM) using a GEMINI-1530 microscope (Carl Zeiss (LEO), Oberkochen, Germany), equipped with an energy dispersive X-ray (EDX) microanalysis system (Oxford Instruments, Abingdon, UK). The nature of metal particles on the catalysts’ surface was evaluated by X-ray diffraction (XRD) using a Bruker D8 Advance diffractometer (Cu K*α* = 1.5406 Å, BRUKER, Rivas-Vaciamadrid, Spain), with 2θ ranging from 10° to 80° at a scan rate of 1°/100 s. The average crystal size (*d*_XRD_) was determined by the Scherrer equation, and iron phases were identified using cards published by the International Centre for Diffraction Data (ICDD/JCPDS). Dispersion and Fe-particle size were also analyzed by high-resolution transmission electron microscopy (HRTEM) using a Titan G2 microscope (FEI, Hillsboro, OR, USA).

X-ray photoelectron spectroscopy (XPS) analysis of the catalysts’ surface was performed using an Axis Ultra-DLD apparatus (Kratos Analytical Ltd., Kyoto, Japan). Survey and multi-region spectra were recorded at C1s, O1s, and Fe2p photoelectron peaks and each spectral region of interest was scanned until good signal-to-noise ratios were obtained. Thermogravimetric analysis (TGA) of the organic supports was performed under N_2_ flow (heating rate of 5 °C/min) using a TGA-50H thermobalance (Mettler-Toledo International Inc., Greifensee, Switzerland) to simulate the carbonization processes, and in air flow to determine the total metal content. The point of zero charge (pH_pzc_) of the materials was determined according to the previously published methodology [56].

### 2.3. Synthetic Wastewater and Experimental Procedure

Experiments were performed with a synthetic effluent comprising five phenolic compounds typically present in real olive mill wastewaters. The concentration/occurrence of each compound was adjusted according to data reported in literature for real OMW as follows: 100 mg/L of tyrosol (2-(4-hydroxyphenyl)ethanol, Sigma-Aldrich, St. Louis, MO, USA), 75 mg/L of gallic (3,4,5-trihydroxybenzoic acid, Alfa Aesar, Haverhill, MA, USA) and caffeic (3,4-dihydroxycinnamic acid, Acros Organics, Geel, Belgium) acids, and 50 mg/L of vanillic (4-hydroxy-3-methoxybenzoic acid, Sigma-Aldrich) and protocatechuic (3,4-dihydroxybenzoic acid, Acros Organics) acids. The compounds were dissolved in distilled water under sonication for 15 min to ensure full dissolution. The chemical characteristics of each compound used in this study are presented in Table S1 of the supplementary information (SI) section. Table 2 highlights the main physicochemical characteristics of the synthetic effluent used in this study as well as the range of values reported for real wastewater: weathered OMW from storage ponds, olives washing waters and OMW from olive oil production (centrifuges).

CWPO runs were performed in triplicate in a 300 mL-capacity cylindrical jacketed batch reactor, under magnetic agitation at ca. 300 rpm (VWR VS-CT magnetic stirrer (VWR, Leuven, Belgium)) and controlled temperature (*T* = 25 °C), recycling water through a model 89202-912 VWR International thermostatic bath (VWR, Leuven, Belgium). The reactor was initially loaded with 150 mL of the synthetic effluent (pH_0_ ~3.8), followed by the catalyst (0.5 g/L) in powder form and the reaction started after the single-step addition of 0.5 mL of H_2_O_2_ (30% *w/v*, VWR Chemicals), yielding an initial concentration of 1.0 g/L. The concentration of hydrogen peroxide selected corresponds to approximately twice the theoretical stoichiometric amount (H_2_O_2_ = 467 mg/L) for complete mineralization of the total organic carbon of the mixture to CO_2_ and H_2_O. The effluent’s pH and temperature were monitored using a WTW Inolab pH-meter and a WTW SenTix 81 combined electrode, respectively. Samples of the effluent were collected at regular intervals and filtered (0.45 µm pore diameter filters) prior to analysis in order to remove the catalyst from solution. Samples were neutralized with NaOH (1 M) and immediately analyzed by high performance liquid chromatography (HPLC), whilst Na_2_SO_3_ was used as quencher of H_2_O_2_ before TOC determinations in order to prevent the homogenous reaction catalyzed by dissolved Fe in the sampling vials. The same procedure was adopted for total phenolic content and TOC determinations of the influent and the procedure repeated on all runs. Pure adsorption of the phenolic compounds was also evaluated for all catalysts in the same operational conditions but in the absence of H_2_O_2_. TOC/TPh removals and H_2_O_2_ consumptions (%) were calculated according to Equation (1):

(1) $$X\left(\%\right)=\frac{C_{0}-C}{C_{0}}\times 100$$

where *X* stands for removal/consumption, *C*_0_ for the initial TOC/TPh/H_2_O_2_ concentration (mg/L) and *C* for the concentration at any given time (mg/L).

The theoretical TOC removal values reported were calculated assuming that all H_2_O_2_ was consumed to completely mineralize organic carbon of each compound up to CO_2_ and H_2_O. For each phenolic compound in the selected mixture, the reaction’s stoichiometry was calculated and the theoretical (mass) rate between TOC and H_2_O_2_ (*R*) is approximately 0.45. The efficiency of H_2_O_2_ consumption (*η*) can then be calculated following Equation (2):

(2) $$\eta \;H_{2}O_{2}\left(\%\right)=\frac{TOC_{removed}\;\left(\frac{mg\;C}{L}\right)}{H_{2}O_{2}{}_{consumed}\left(\frac{mg}{L}\right)\times R}\times 100.$$

### 2.4. Analytical Methods

Concentration of individual phenolic compounds (both for the influent and the effluent samples) was determined by HPLC with diode-array detector (HPLC-DAD), using a Hitachi Elite LaChrom apparatus (Hitachi, Tokyo, Japan), consisting in an L-2310 pump, L-2200 auto-sampler and L-2455 DAD. The chromatographic separation was achieved by a Purospher STAR RP-18 column (240 mm × 4 mm, 5 µm) at 50 °C, using a mobile phase composed of 70% (*v/v*) of ultra-pure H_2_O slightly acidified with orthophosphoric acid and 30% (*v/v*) of methanol (≥99.8%, Fischer Chemicals, Zurich, Switzerland), at isocratic conditions, with a flow rate of 1 mL/min. The injection volume was 20 µL and the spectra were recorded at 280 nm. Standards calibration curves were previously prepared for the identification and quantification of the parent phenolic compounds present in the mixture. 

Total organic carbon (mg C/L) was measured using a TC/TOC Shimadzu TOC-L apparatus, while the concentration of H_2_O_2_ in solution was determined by the Sellers colorimetric method [60] in virtue of its fast and accurate results. Toxicity values were obtained following the standard DIN/EN/ISO 11348-3, where the bioluminescence inhibition of *Vibrio fischeri* bacteria is assessed; a Microtox model 500 apparatus (Modern Water, London, UK) was used and the bacteria’s bioluminescence evaluated at different contact times (5, 15 and 30 min.) at 15 °C. Iron leaching from the catalysts was determined by flame atomic absorption spectrometry using a model 939/959 AAS UNICAM spectrophotometer (Thermo Fischer Scientific, Waltham, MA, USA) (Standard Methods—Method 3111 B). 

## 3. Results and Discussion

### 3.1. Textural and Chemical Characterization

As mentioned in the previous section, two solid residues from different steps of the olive oil production were selected to develop Fenton catalysts’ supports: sawdust (SD) from the olive tree pruning and olive stones (OS) obtained after the oil’s extraction process. The objective is, evidently, the valorization and integration of such waste/cheap materials in a more efficient and clean olive oil production, by facilitating the treatment and reuse of water and reducing the pollution load of the produced effluents in the same agro-industrial activity sector.

**Figure 1:**
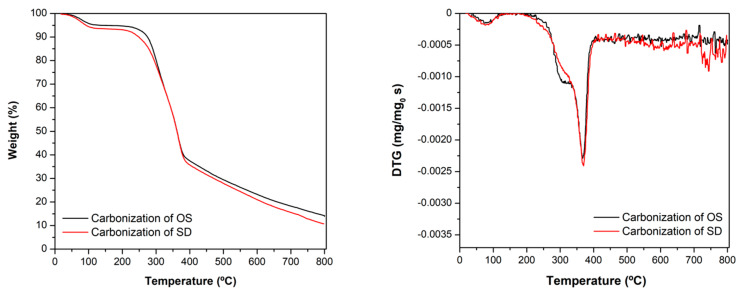
TG-DTG profiles for the carbonization of OS and SD residues.

Both lignocellulosic residues were carbonized in order to increase their chemical stability and development of porous textures. Initially, the carbonization process was simulated by TGA in order to fit experimental conditions of samples’ preparation. TG-DTG (differential thermogravimetric analysis) curves obtained in each case are compared in Figure 1. It is noteworthy the good coincidence of pyrolysis curves, denoting a similar chemical structure and stability of both raw materials. The carbonization process of lignocellulosic materials is complex [61,62,63] and it starts with the decomposition of hemicellulose, typically occurring between 200–290 °C with the formation of volatile compounds. In this case, however, it is observed that the first weight loss process occurs at around 100 °C, then weight remains nearly constant up to 250 °C. Thus, probably only dehydration occurs in this temperature range. The main carbonization step takes place between 250–380 °C, associated to the cellulose decomposition that is the main component in both residues. Afterwards, carbonization continues with increasing temperature, although at a slower and nearly constant rate, due to the greater thermal stability of lignin regarding cellulose. Those processes are more clearly observed in the DTG profiles, corresponding respectively to the small minimum at ca. 100 °C, the shoulder around 300 °C and the maximum carbonization rate at ca. 350 °C. Finally, the DTG curves remain practically constant at high temperature because, as commented, the observed lignin decomposition rate is nearly constant. In both cases the weight loss (WL) up to 800 °C was around 85 wt.%. The ash content determined after burning a sample fraction in air was also similar in both cases (ca. 2.5 wt.%). The experimental conditions of OSC and SDC physical activation, as well as the chemical activation of OS, were fitted in order to have a similar activation degree, with an activated carbon yield between 14–16 wt.%.

**Figure 2:**
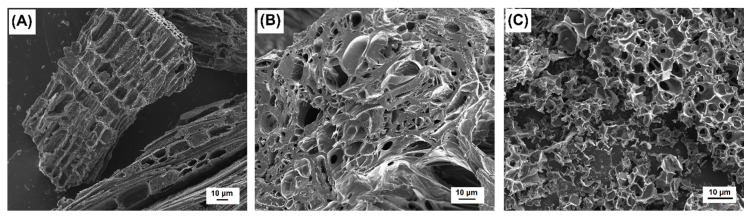
High-resolution scanning electron microscopy (HRSEM) images of: (**A**) SDC-AC, (**B**) OSC-AC, and (**C**) OS-AC/KOH supports (without Fe).

The morphology of the samples was studied by HRSEM (Figure 2). The cellular structure of wood is visible in the physically-activated carbon obtained from SDC (Figure 2A) with long, wide, and parallel channels aligned in the direction of the tree’s growth. Although the particles of AC obtained from OSC also exhibit a highly porous structure (Figure 2B), closed vesicles are observed and the channels are clearly narrower than the ones in SDC-AC and not so clearly aligned, thus, most compact particles are observed. After chemical activation, the cellular structure of OS is strongly damaged and the tangled combination of crisscrossed channels leads to the foam-like aspect (Figure 2C) instead of regular channels.

Samples’ porosity was determined by N_2_ and CO_2_-adsorption isotherms and the textural characterization results are compiled in Table 3. Both prepared activated samples and commercial materials present predominantly N_2_-adsorption isotherms of type I–IV, characteristic of microporous/mesoporous materials. The N_2_-adsorption isotherms of all supports and their corresponding catalysts presented hysteresis loop, indicating some contribution of mesopores in their porous structure (Figure S1). N_2_-adsorption allows to determine the total porosity of samples in the absence of diffusional restriction, while CO_2_ is used to characterize the narrowest micropores (ultramicropores with diameter <0.7 nm), where the accessibility of N_2_ at 77 K can be limited. However, CO_2_ is only adsorbed into the microporosity because of the higher saturation pressure (*P*_0_) at 273 K. In this case, only OSC and SDC biochars present a close microporosity inaccessible to N_2_, thus the micropore volume *W*_0_(N_2_) < *W*_0_(CO_2_) and the surface area (*S_BET_*) is low in both cases (around 100 m^2^/g). On the contrary, for both synthesized or commercial activated carbon samples, *W*_0_(N_2_) > W_0_(CO_2_), denoting that physical activation develops the porosity, avoiding diffusional restriction of N_2_ and also favoring the adsorption in larger micropores. Nonetheless, the narrowest microporosity—*W*_0_(CO_2_)—varies only slightly. Activation leads to a significant increase of the surface area, reaching a value close to 800 m^2^/g for OSC-AC. When comparing OSC-AC and SDC-AC supports it is noticeable the similarities in total porosity (*V_T_* = 0.39 cm^3^/g). Nonetheless, the formation of more opened porosity, wider micropores, and greater mesoporosity is favored in the latter, and thus, in spite of the similar total pore volume, the surface area value is significantly smaller (565 m^2^/g). The highest porosity is obtained after chemical activation of OS, which strongly favors the formation of a large micropore volume. This well-developed microporosity leads to higher surface area values (ca. 1000 m^2^/g). Both commercial activated carbons are also eminently microporous samples: N sample shows similar porous characteristics as compared to OS-AC/KOH support, and sample M is comparable with OSC-AC.

**Figure 3:**
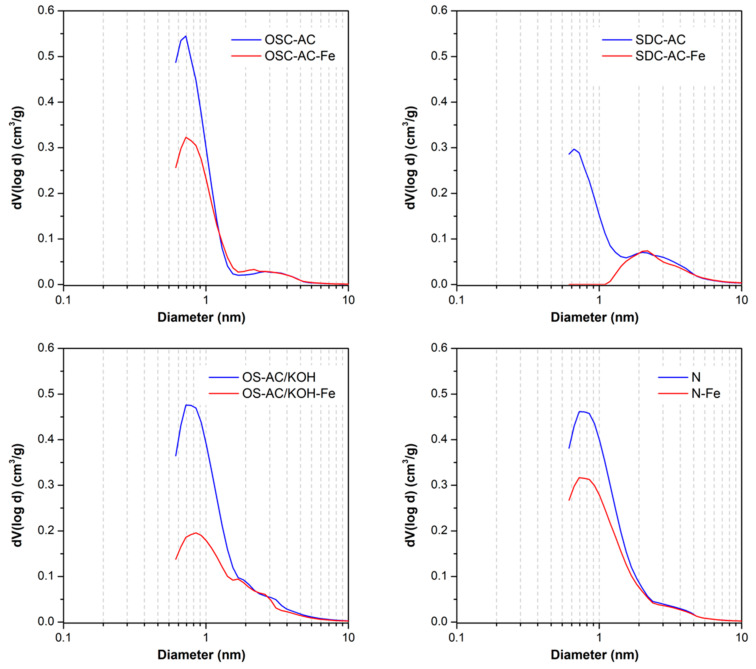
Pore size distribution obtained by Quenched Solid Density Functional Theory (QSDFT) applied to N_2_-adsorption isotherms for selected activated carbon supports and Fe-derivative catalysts: effect of Fe-impregnation.

After Fe-impregnation, all catalysts present a smaller porosity than the corresponding supports because Fe-nanoparticles are partially blocking or occupying this porosity (Figure 3). Such blockage affects mainly the microporosity range (which is blocked and not accessible to N_2_), thus increasing the mean pore size *L*_0_ on all catalysts regarding their corresponding supports. Fe-nanoparticles seem to be located mainly on the larger micropores, but the porosity larger than 1.5–2.0 nm is preserved (Figure 3). This fact is corroborated by the strong decrease of W_0_ values after impregnation of supports with large micropores, particularly for OS-AC/KOH and SDC-AC. Due to the low micropore volume and large micropore size of SDC-AC, microporosity is practically blocked in the obtained SDC-AC-Fe, thus showing the lowest *S*_BET_ of catalysts derived from activated supports. Consequently, the support’s porous attributes also define the characteristics of the Fe-active phase in the catalysts derivatives. The acid/basic character of the samples is discussed on the basis of their pH_pzc_ values (Table 3). The pH_pzc_ of the supports range from 7.0 to 11.6, being consequently basic materials. However, after impregnation with the Fe-active phase, catalysts become acidic materials.

**Figure 4:**
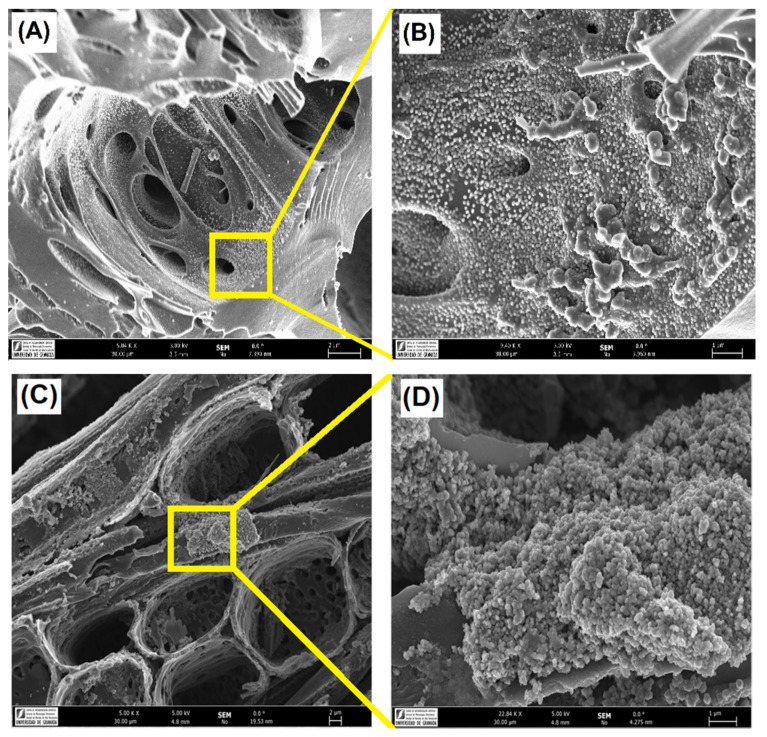
Morphology of: (**A**) OSC-AC-Fe (**C**) and SDC-AC-Fe; and (**B**,**D**) detail of Fe-nanoparticles coating the carbon surface.

**Figure 5:**
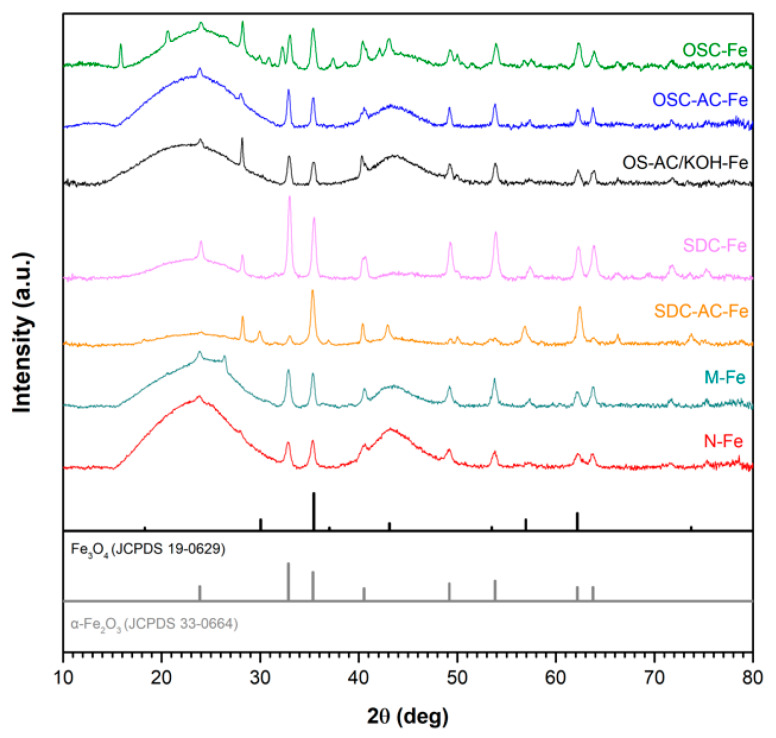
X-ray diffraction (XRD) patterns of the catalysts tested; standard patterns of Fe_3_O_4_ and *α*-Fe_2_O_3_ (JCPDS cards no. 19-0629 and 33-0664, respectively) are also presented.

**Figure 6:**
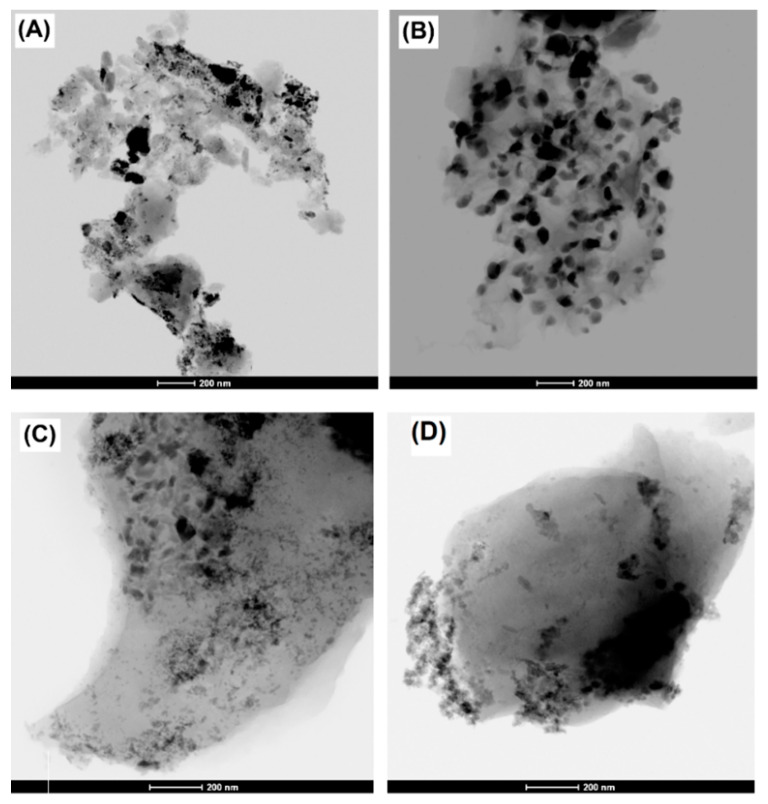
High-resolution transmission electron microscopy (HRTEM) images of (**A**) OSC-AC-Fe, (**B**) OS-AC/KOH-Fe, (**C**) SDC-AC-Fe, and (**D**) N-Fe catalysts.

Nature and dispersion of the Fe-active phase was analyzed by the combination of HRSEM and HRTEM-EDX analysis, XRD and XPS techniques. After impregnation, as expected, catalysts maintain the same morphology as previously described, but it is clearly observed the formation of a high concentration of new nanoparticles coating the carbon surface (Figure 4). The XRD patterns presented in Figure 5 point out that iron nanoparticles are transformed into oxides after pretreatment of the impregnated samples. Basically, hematite, α-Fe_2_O_3_ (JCPDS card no. 79-0149) is detected, although magnetite, Fe_3_O_4_ (JCPDS card no. 79-0149) was also identified in a minor proportion. X-ray diffraction patterns also show two broad peaks for all catalysts at 2*θ* values around 26° and 44° (JCPDS card no. 89–8487), corresponding to the (002) and (101) diffraction peaks of graphite, respectively. No significant differences were noticed between the nanocrystal sizes of such Fe-phases among the catalysts, being in all cases between 19 and 23 nm (as obtained from the Scherrer equation). Although Fe-particles were detected by HRSEM on the samples’ surface, it is also expected the formation of smaller nanoparticles which can be located into the porosity, as previously denoted by textural characterization. HRTEM images (Figure 6) show heterogeneity in both size and distribution of iron particles. Differences are based on the distinct interactions of the aqueous precursor’s solutions with the supports. The distribution of Fe-nanoparticles on physically activated biochars and commercial *N*-support is very heterogeneous, with particle sizes ranging from very small particles of a few nanometers to large ones exceeding 100 nm, responsible for the XRD peaks. Additionally, in some cases, the morphology of the Fe-particles changed from dense units with high contract to “cloud” shape structures. In the case of chemically activated OS, however, the distribution, shape and good contrast of Fe-particle size (at around 50 nm) are quite homogeneous.

**Figure 7:**
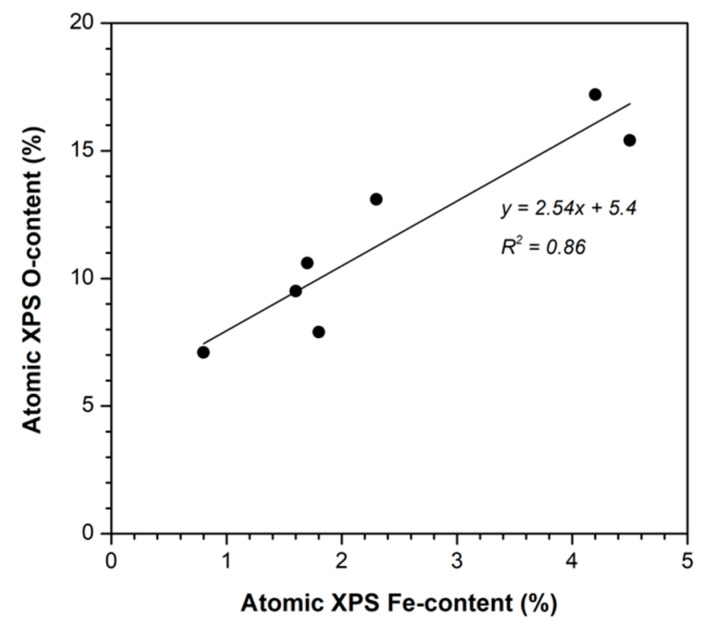
Relationship between the surface’s Fe and O atomic contents (%) for all the prepared catalysts.

The catalysts’ surface composition obtained by XPS is summarized in Table 4. The surface Fe-content is higher in the SD series than in the OS one, and increased from carbonized to activated samples on both situations, thus also confirming that the opened porosity enhances the Fe-dispersion and/or the localization of the Fe-nanoparticles in a more external surface accessible to the XPS analysis. The surface oxygen content increased with the Fe-content (Figure 7), though the slope of this line is around 2.5, greater than the O/Fe ratio for any possible oxides (1.5 for Fe_2_O_3_ and 1.33 for Fe_3_O_4_), because the determined oxygen content evidently included the oxygenated surface groups of the carbon supports. The analysis of a catalyst after an oxidative reaction (denoted as “OSC-AC-Fe used” in Table 4) shows a similar Fe-content to the analogous “fresh” one, denoting that the Fe-leaching is not significant in this sample. Nevertheless, the oxygen content strongly increased, indicating the oxidation of the carbon surface by H_2_O_2_ during the reaction or the adsorption of pollutants (or their oxidation intermediates) on the catalyst surface. In fact, this increased oxygen content in activated carbons was also determined when used in catalytic wet air oxidation (CWAO) of aniline [64].

**Figure 8:**
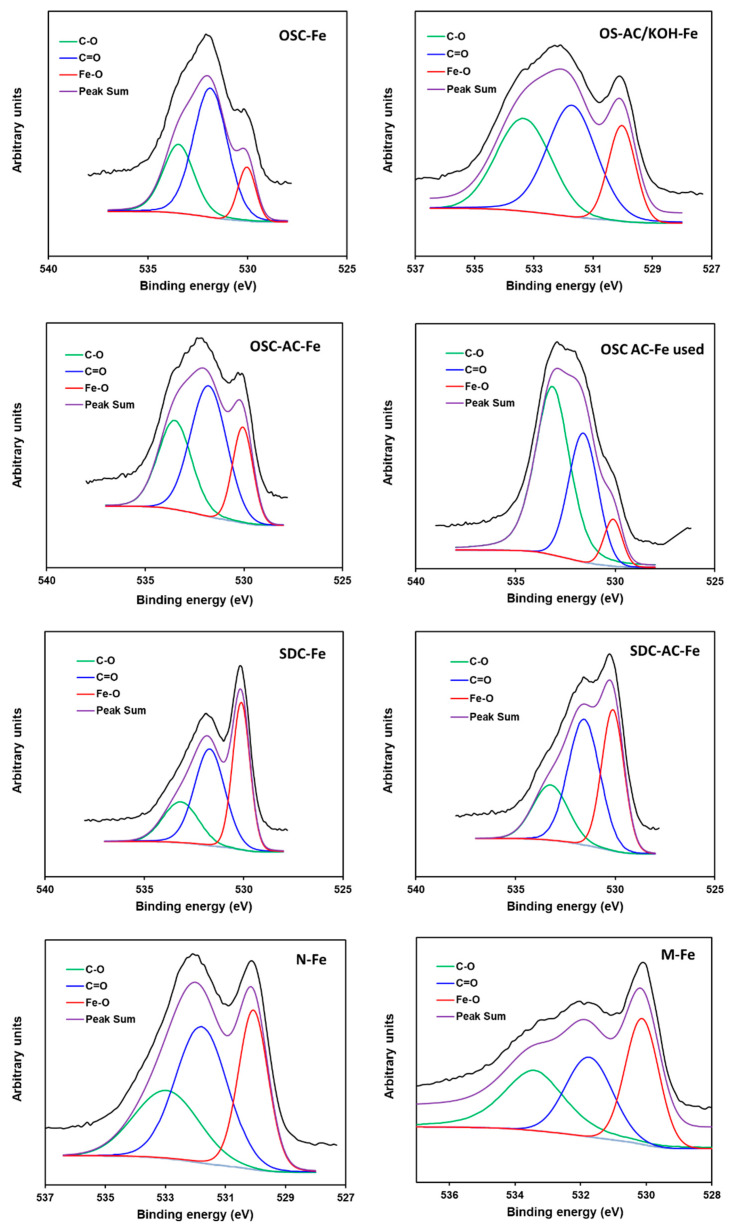
O1s XPS spectral region of the different catalysts.

The different high resolution XPS spectral regions (C1s, O1s, and Fe2p) were treated to obtain the surface chemistry composition and results are summarized in Table 4 and Table 5. The Cs1 spectra were fitted using four components [65,66]. The first component at 284.6 eV is used as reference, and is due to aliphatic and aromatic C–C bonds, while the rest of components were assigned to carbon forming oxygen functionalities: single C–O bonds (285.7 eV), double C=O bonds (286.8 eV), and O=C–OH carboxylic structures (288.4 eV). The proportion of carbon linked to oxygen increased from chars to their derivative activated carbons due to the surface oxidation by CO_2_-treatment (e.g., C–C contribution at 284.6 eV decreased from 71 to 66% in OSC-Fe and OSC-AC-Fe, respectively—Figure S2). The oxygen content (Table 4) also increased but, as previously commented, due to the greater Fe-surface content. The distribution of oxygenated surface groups (OSG) is more clearly pointed out by analyzing the O1s spectral region (Figure 8, Table 5). In this case, three components were used, being the first one located at 530.1 eV and assigned to the Fe–O bonds [67,68], clearly differentiated in the O1s profile by important shoulder at this binding energy that in some cases increased to an independent maximum The other two components located at 531.6 and 533.2 eV are assigned to the OSG on the carbon surface, double C=O and C–O bonds, respectively [65,66]. Broadening of the peak at around 531 eV is suggested to be due to the interactions of the Fe-nanoparticles with the OSG of the carbon supports, forming Fe–O–C bonds [68,69].

As previously mentioned, the surface composition determined by XPS indicated that Fe-leaching was not significant for the OSC-AC-Fe catalyst during the reaction. Nevertheless, the oxygen content strongly increases, and the analyses of oxygen nature (Table 5, Figure 8) pointed out that this was due to the oxygen linked to carbon (either to the carbon supports or the adsorbed species). The Fe–O ratio decreased from 20% to 8%, highly increasing the proportion of the C–O bonds, likely indicating the adsorption of phenols (C–OH) and their derivatives. It is noteworthy the higher contribution of oxygen linked to iron (Fe-O) in catalysts supported on commercial and SD-derived supports regarding those supported on OS-derived supports, clearly related also with the higher surface Fe-content of these samples (Table 4), induced by a more opened porosity (as previously stated).

**Figure 9:**
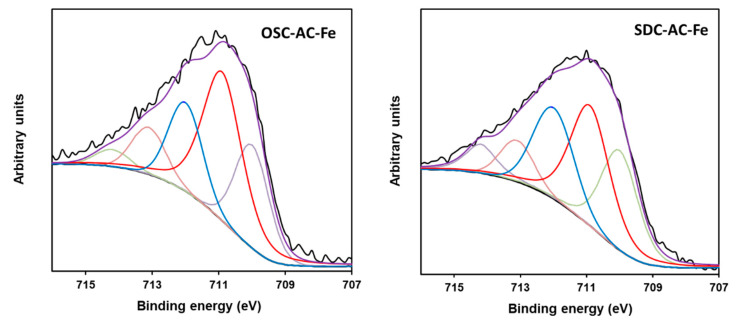
Fitting of the 2p_3/2_ spectral region of OSC-AC-Fe and SDC-AC-Fe samples.

To determine the chemical state of the surface iron, the Fe2p spectral region was fitted with five components (Figure 9), according to the procedure of McIntyre and Zetaruk [70]. This region presents a similar profile for all catalysts. The peak maximum in this region is fixed at 710.9 eV with a full width half maximum (FWHM) value of 1.4 eV, that together with the Gupta and Sen (GS) multiplets at 711.9, 713.0, and 714.1 eV, with FWHM = 1.2 eV, were assigned to Fe^3+^ species [67,68,71], whilst the component located at 709.9 eV corresponds to the Fe^2+^ ones. Except for the OSC-Fe catalyst, that exhibits a lower Fe^2+^ content, the remaining samples present a quite similar Fe^2+^/Fe^3+^ ratio, which is in agreement with the XRD results, showing the preferential formation of Fe_2_O_3_-particles. Nevertheless, it is noteworthy that the Fe^2+^ content is in general significantly high, suggesting the possibility of the formation of non-crystalline phases of partially reduced oxides, including magnetite (Fe_3_O_4_), detected as minority phase by XRD.

### 3.2. Treatment of Simulated OMW

**Figure 10:**
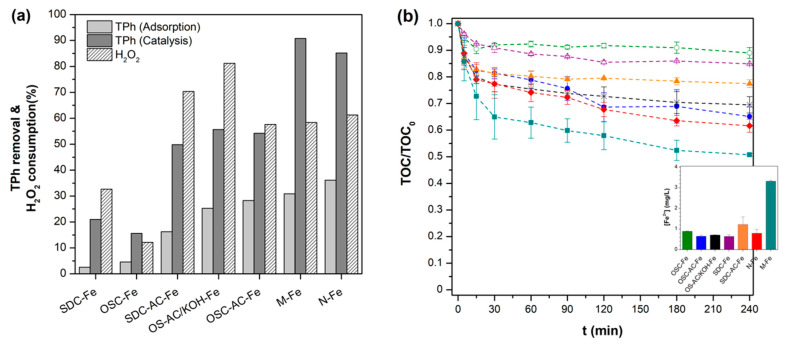
(**a**) Total phenols (TPh) removal by adsorption and catalytic processes, and H_2_O_2_ consumption for each catalyst after 4 h of reaction and (**b**) evolution of TOC conversion as a function of reaction time for catalytic wet peroxide oxidation (CWPO) experiments (inset: mean Fe-leaching values after the reaction).

The CWPO of OMW was studied. In order to estimate the influence of the adsorptive character of the catalysts in the CWPO process, experiments in the absence of H_2_O_2_ were carried out in such a way that the pollutant removal obtained in each case is evidently only due to adsorption phenomena. The performance of the catalysts in the removal of the total phenolic content (TPh) by adsorption and catalytic processes is summarized in Figure 10a.

Using catalysts prepared on the biochar supports OSC and SDC results in a low adsorption of pollutants from the initial mixture (bellow 5% of total phenolic content). When using catalysts prepared from activated supports, adsorption is still low for the SD-AC-Fe catalyst because, as previously shown, microporosity is practically blocked after Fe incorporation, resulting in a TPh removal of 16% after 240 min. Using OS-AC/KOH-Fe and OSC-AC-Fe, higher removals were achieved by adsorption (25% and 28%, respectively) because both catalysts present a similar surface area (ca. 550 m^2^/g—Table 3). In the case of the commercial supports based catalysts, both M-Fe and N-Fe achieved the highest adsorptive performance of pollutants after 4 h, with ca. 31% and 36%, respectively. Individual contaminants’ removal by adsorption (*C*/*C*_0_) over time for each catalyst is reported in Figure S3 of SI.

Regarding CWPO experiments, it was demonstrated that in the absence of any catalyst and under the same experimental conditions reported hereafter, the degradation of contaminants by H_2_O_2_ alone is negligible (see Figure S4 of Supplementary Information), due to the low oxidation potential of hydrogen peroxide. Fe-catalysts prepared from biochar OSC and SDC supports exhibited the lowest removal of TPh (Figure 10a). Nonetheless, while OSC-Fe is slightly more effective as adsorbent, SDC-Fe is more active in CWPO, associated to the more developed porosity of the first (Table 3), and the higher iron surface-content of the second (Table 4). This points out the importance of a developed porosity and accessible Fe-nanoparticles. In this sense, the catalytic performance also improved using activated supports because physical or chemical activation of residues improves both factors. Therefore, the better performance of catalysts supported on commercial activated carbons is due to their higher surface area and surface Fe-content.

While TPh removal values show good coincidence with TOC ones in adsorption experiments, both parameters present differences in the oxidation runs due to the formation/adsorption of intermediate/final reaction products. In Figure 10b, catalysts’ performance over time in the CWPO process are compared in terms of mineralization. Although the same order of activity is generally observed, being M-Fe catalyst the most active with ca. 50% total reduction in TOC, it is observed a very similar performance of OSC-AC-Fe and OS-AC/KOH-Fe catalysts in respect to the N-Fe one. As observed in Figure 10a, the adsorptive capacity of such materials is quite similar, while TPh removals achieved with N-Fe are considerably higher, suggesting the formation of a significant amount of intermediates that remain in solution during the synthetic OMW treatment with such catalyst.

The concentration of Fe dissolved in solution (in mg/L) after the CWPO runs was also analyzed and the mean values for each catalyst are reported in the inset graph of Figure 10b. The Fe-leaching from M-Fe is around four times greater than the values obtained when Fe is supported on biochars or activated carbons from olive industry’s residues. Thus, despite M-Fe seems to be at a glance the most active material of this series, it can be related to the higher activity of Fe in solution, where it can work as a homogeneous rather than heterogeneous catalyst. The lack of stability is evidently a significant hindrance regarding the catalyst’s life and possible reuse. Moreover, the leaching of metal ions to the solution has to be avoided to prevent additional contamination and respect environmental legislations (maximum of 2 mg/L in EU directives [72]). With the exception of the aforementioned material, leaching of iron was typically below 1 mg/L for the remaining materials, which corresponds to <1.5% of the initial theoretical Fe load in the catalysts, indicating a strong anchorage of the metal to the carbon surface. To check the influence of the homogeneous Fenton process in the mineralization efficiency, two experiments in homogeneous phase were performed using Fe concentrations corresponding to the average minimum and the maximum observed for this series of catalysts. It was found that 1 mg/L of Fe is only able to remove ca. 6% of TOC, whilst 3.5 mg/L result in ca. 20% mineralization after 240 min (results not shown).

**Figure 11:**
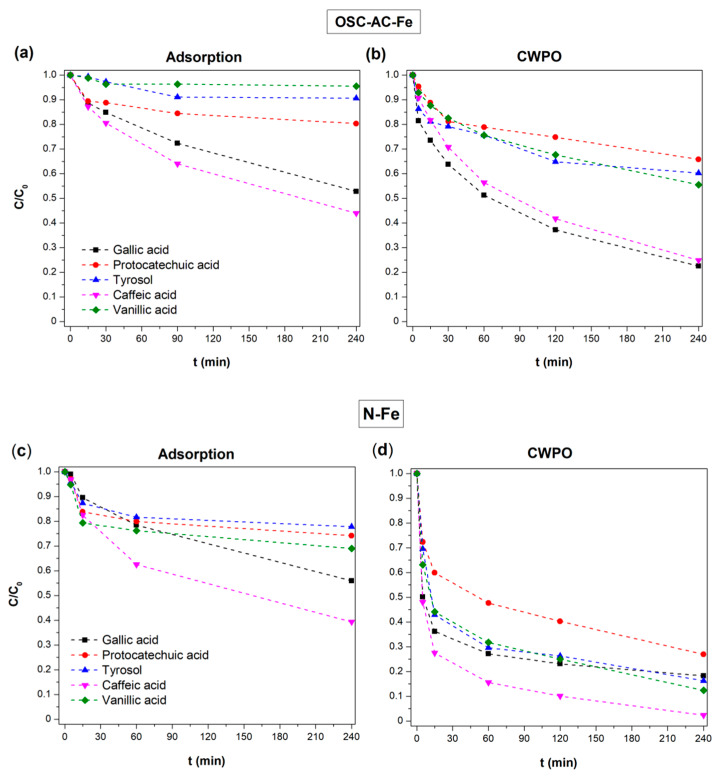
Comparison of phenolic compounds’ removal by adsorption or CWPO using OSC-AC-Fe and N-Fe catalysts.

The removal of each phenolic compound along adsorption and CWPO processes was followed by HPLC—full details are provided in Figures S3 and S5 of the Supplementary Information, respectively. Figure 11 shows, as an example, the comparison of performances for synthesized OSC-AC-Fe and N-Fe catalysts in adsorptive and catalytic experiments. It is observed the occurrence of preferential adsorption of caffeic and gallic acids regarding the tyrosol, vanillic or protocatechuic acids. This behavior is common for all the catalysts prepared and tested in this study. After 240 min of CWPO reaction, both catalysts showed a similar TOC/TOC_0_ ratio (0.65 and 0.62, respectively), although the oxidation of each compound is evidently distinct. Results also showed that the oxidation of each compound is related with the previous adsorption process, highlighting the dependence on the pollutant/catalyst surface interaction, as they follow the same degradation order.

**Figure 12:**
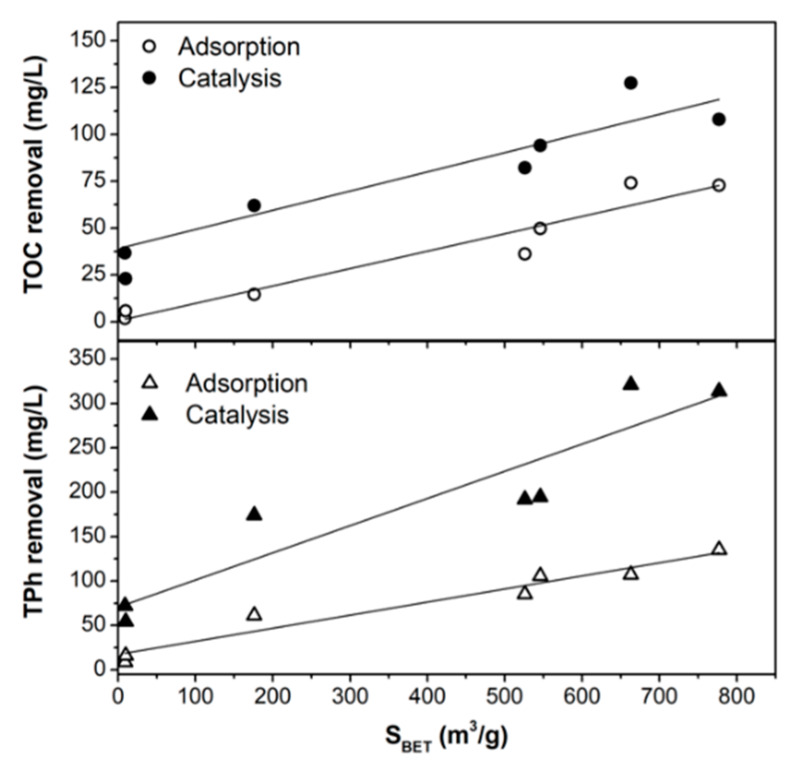
Influence of the specific surface area (*S*_BET_) on the TPh and TOC removals by adsorption and catalysis (in mg/L).

The interactions of phenolic compounds with the catalysts surface are dependent on the catalysts porous structure (determining diffusion, adsorption rate, and adsorption capacity) but also on the chemical interactions between pollutants and the adsorbent’s surface, thus, on the nature and distribution of the surface chemical groups on the catalysts and the chemical structure of phenols. The main role of the porous texture of the catalysts on their adsorptive and catalytic performance is highlighted in Figure 12.

In previous works, it was demonstrated that carbon materials’ adsorption of phenols is related to parameters such as their solubility in water [73]. Nonetheless, results in this study show that caffeic and vanillic acids, which present similar solubility (Table S1), exhibited opposite adsorption behavior (Figure 11). Also, the affinity of different phenols by the carbon’s surface varies according to the nature of the aromatic rings substituents [74]. Adsorption of phenols is favored by the basic character of the adsorbent and by the withdrawing effect of substituents. Electron withdrawing groups from the aromatic rings such as halogens (–X) or nitrites (–NO_2_) enhance adsorption regarding electron donor groups such as hydroxyl (–OH) or amino (–NH_2_) substituents [74]. Different positions were therefore activated on the aromatic rings depending on the substituents nature and position, also determining the reactivity and degradation mechanism in Fenton reactions [75]. Iron species can also induce various chelation degrees with the different phenolic acids [76]. Results showed that hydroxycinnamic acids (in our case the caffeic acid) are typically better ligands for iron than the hydroxybenzoic ones (gallic, vanillic, or protocatechuic acids). The ethylene group between the aromatic ring and the carboxylic group in hydroxycinnamic acids influence not only the chelating activity, but can also work as a scavenger, preventing free radical reactions. Regarding the hydroxybenzoic acids, the number and position of hydroxyl groups also influences the chelating effect and the galloyl moiety improving chelation regarding catechol groups, thus the gallic acid is a best chelating agent than the protocatechuic one. The stability of the Fe-phenolic acid complexes was compared by determining their binding constants [76], with the following order being obtained: protocatechuic acid < gallic acid < caffeic acid. Our experimental data showed a preferential removal of caffeic and gallic acids, which can be related with this effect. Nevertheless, specific experiments of characterization of used samples still need to be performed in order to clarify this aspect.

Once the phenolic compounds are adsorbed, the oxidation also depends on the reactivity of each molecule. The electrophilic attack of hydroxyl radicals to phenols’ aromatic rings is facilitated by the presence of electron donating groups (EDG), explaining the fact that oxidation of gallic acid (with three EDG) is always higher than the remaining benzoic acid derivatives (vanillic and protocatechuic acids). Previous studies [77] also showed that degradation of cinnamic acid derivatives is generally faster than benzoic acid derivatives, which justifies the high reactivity of the caffeic acid (containing two reactive groups in its structure) observed in this study. S. Azabou et al. [78] reported on the catalytic photo-oxidation of a phenolic mixture containing, among others, tyrosol and vanillic and caffeic acids. Under the experimental conditions employed in that work, the degradation yield of caffeic acid (86%) was higher than vanillic acid (50%) and tyrosol (31%), also proceeding at a faster oxidation rate than the last two, which is also observed as a trend in our study.

The degradation profiles of contaminants by Fe-AC based catalysts (Figure 10b, Figure 11b,d), also point to a two-stage process, characteristic of Fenton-related processes, with a very fast initial oxidation of organics up to *t* = 15–30 min followed by a slower degradation rate, likely associated to the Fe regeneration cycle [79]. The catalytic oxidation of the phenolic solution is accompanied by the formation of reaction intermediates, namely carboxylic acids and also their complexes with iron ions [47], in greater or lesser extent depending on the physicochemical properties of the catalysts used. Such reaction intermediates are commonly responsible for the drop of the solution pH [57], which was also observed on all runs (from pH_0_ ~3.8 to 3.0–3.4, depending on the catalyst—data not shown). Oxalic acid was identified as the main reaction intermediate in solution after the treatment, while oxamic and maleic acids were also identified in some cases but in a smaller proportion (data not shown).

**Figure 13:**
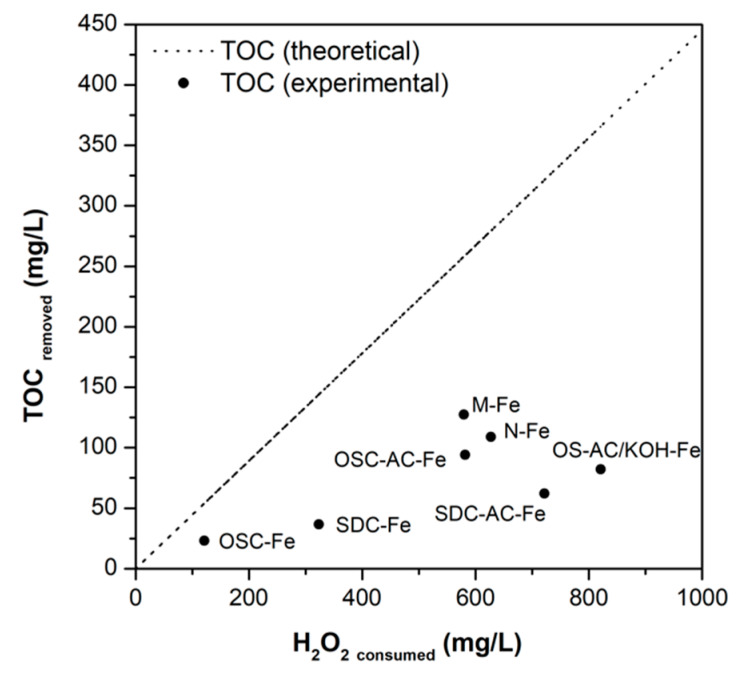
H_2_O_2_ consumed vs. experimental (and theoretical) TOC removals for each catalyst.

High TOC removal and efficient H_2_O_2_ conversions are indispensable for assessing any catalyst’s performance in the CWPO process. Conversions of H_2_O_2_ (%) are displayed in Figure 10a, while the oxidant consumption (mg/L) for each catalysts vs. the correspondent TOC removal is plotted in Figure 13.

The plot of H_2_O_2_ conversion vs. TOC removal shows that OSC-AC-Fe presents one of the highest oxidant use efficiencies (*η* = 36%) among the catalysts obtained using supports prepared from activated residues (the highest was obtained using OSC-Fe, but mineralization is very low). The performance of this catalyst is similar to those obtained using the commercial support N-Fe (*η* = 39%) but lower than M-Fe (*η* = 49%). In comparison, the M-Fe catalyst presents a surface area and surface iron content similar to OS-AC/KOH-Fe, but a narrower microporosity and more heterogeneous Fe-particles distribution. Compared with SDC-AC-Fe, OSC-AC-Fe exhibits a larger surface area but smaller surface iron content (Table 3 and Table 4). Owning to the complexity and diversity of processes occurring simultaneously, but also on the physicochemical heterogeneity among the materials tested, it is virtually impossible to correlate them with all the results obtained. In that sense, the lower H_2_O_2_ consumption efficiencies of some materials may be related to the fast scavenging of ^•^OH radicals and/or non-selective decomposition of the oxidant by bulk Fe-oxides and hydroxides, as already reported by other authors [80]. Moreover, it is also well-known the ability of carbon materials to catalytically decompose H_2_O_2_ molecules via different routes, even in the absence of any transition metal [81], for which the real yield of ^•^OH radicals generated may vary according to the intrinsic properties of each material.

**Figure 14:**
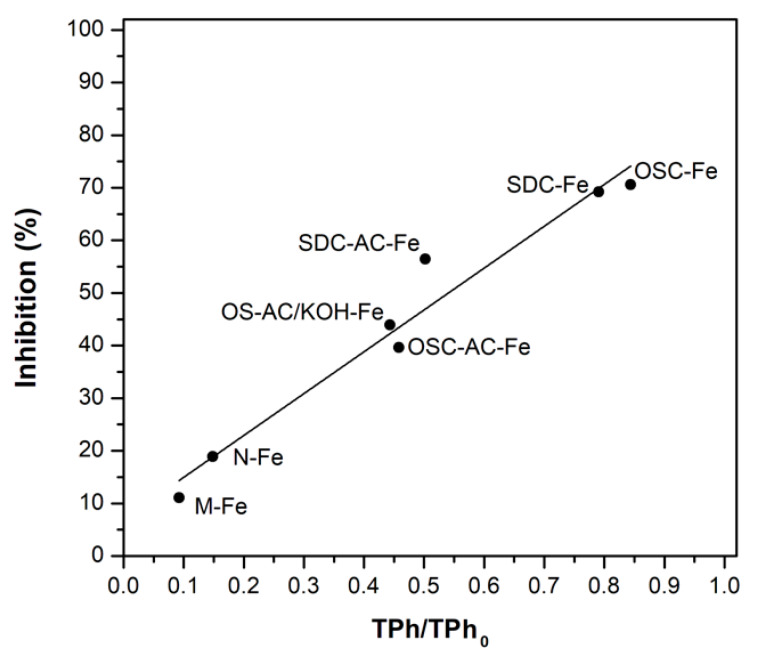
TPh/TPh_0_ vs. inhibition (%) of the *V. fischeri* bacteria after 30 min of contact time with the different solutions after CWPO.

The level of toxicity and biodegradability of OMW is commonly related to the amount of TPh present in solution [58]. Toxicity values were evaluated following the inhibition (%) caused to the bioluminescent *V. fischeri* bacteria when in contact with the phenolic solutions (contact times of 5, 15, and 30 min). The initial untreated effluent presented 99% bioluminescence inhibition after only 5 min of contact time, clearly denoting its toxic character towards this bacterium. A relation between the amount of TPh removed from each solution (by each catalyst) after the CWPO process and *V. fischeri* inhibition (after 30 min of contact time) was established (Figure 14). As anticipated, the highest toxicity reduction was achieved by N-Fe and M-Fe catalysts (only 19% and 11% bioluminescence inhibition, respectively), related to the higher TPh oxidation achieved by those catalysts. Nonetheless, more than 60% of the initial toxic character of the phenolic solution was eliminated using Fe-catalysts prepared from physically activated olive stones (OSC-AC-Fe), whilst 56% and 44% reductions were achieved by OS-AC/KOH-Fe and SDC-AC-Fe catalysts, respectively.

As previously mentioned, the formation of reaction intermediates, potentially more refractory than the original ones, is a major drawback related to AOP applications. However, the correlation obtained between the toxicity values of the treated effluent samples and the removal of initial pollutants clearly suggest that, despite the formation of intermediates, they exhibit a nontoxic behavior or, at least, less toxic than the original pollutants.

### 3.3. Further Considerations

This study presents an approach with double interest for the olive oil sector, combining the valorization and integration of solid residues in the management of wastewaters generated by this industry, resulting in a more environmentally friendly process. However, it is primarily focused in one particular category of refractory pollutants that comprise the overly-complex physicochemical character of olive mill wastewaters. The reduction of the phenolic content of OMW (and thus its overall toxic character), whilst unveiling the specific interaction of those contaminants with the synthetized materials, is the first step to understand and improve catalysts’ synthesis for such applications.

As reported, the proposed process is clearly able to degrade a broad range of phenolic contaminants and thus reduce the toxicity of the treated effluent. Evidently, the application of the proposed process to more complex wastewater matrixes would require the optimization of the operational conditions—e.g., pH, temperature, and particularly H_2_O_2_ and catalyst concentrations—depending on the specific treatment requirements. In fact, the efficiency of most AOPs is often improved by the integration of pre- and post-treatment units in a multi-step treatment scheme. Simple but finely tuned physicochemical steps, such as coagulation/flocculation or filtration processes, will considerably reduce costs associated to subsequent treatment stages and improve the overall efficiency of the process. Likewise, the oxidation of recalcitrant compounds and overall mineralization of organics would favor the action of microorganisms in a subsequent (downstream) biological treatment unit, or even allow its discharge on crops or wastewater collectors (provided the compliance with local discharge environmental regulations).

In that sense, and despite the limitations of CWPO processes related to catalysts’ deactivation through Fe-leaching and costs associated with H_2_O_2_ consumption (that will depend on the degree of oxidation required), the equipment simplicity, easy-to-handle reagents and operational conditions required (atmospheric pressure and room temperature) clearly favors the application of CWPO when compared to more energy demanding processes (e.g., ozonation and catalytic wet air oxidation) or those limited by the dark color of OMW (e.g., photocatalysis).

In order to enhance the stability of the proposed materials and improve the process’s efficiency, ongoing studies are focused on the catalysts synthesis, as to improve the Fe-dispersion and anchorage to the prepared supports, as well as their application to more complex wastewater matrixes, such as pre-treated or diluted real OMW. Then, optimized materials will be tested under a wider range of experimental conditions, including different reactor configurations, particularly operating under conditions closer to continuous operation (e.g., packed-bed column and/or continuous stirred tank reactors). If efficient materials are developed, a detailed techno-economic analysis should be done, and effluents characteristics compared with legislated standards.

## 4. Conclusions

Fe-catalysts were prepared by direct impregnation of biochars and activated carbons obtained from sawdust (tree pruning) and olive stones by combining carbonization with chemical or physical activation processes to yield a similar activation degree. The physicochemical properties of these materials depend on the raw residue and experimental conditions of synthesis. Thus, the synthetized Fe-AC catalysts are essentially microporous materials with BET surface areas ranging from 176–777 m^2^/g and with a high concentration of more or less spherical Fe-nanoparticles (mainly as *α*-Fe_2_O_3_), covering the carbonaceous surface of the materials, partially blocking or occupying the microporous structure, in particular for supports obtained from sawdust pruning.

Adsorptive and catalytic screening tests of a polyphenolic solution simulating the OMW showed the importance of the porous structure of the materials, which controls the accessibility of phenolic compounds to Fe-active sites and therefore not only the adsorption capacity of the pollutants, but also the catalytic performance. Both TPh removal and the mineralization degree (TOC) increased linearly with increasing the surface area of the catalysts. Thus, ACs showed an enhanced adsorptive and catalytic behaviors regarding the corresponding biochars.

On the other hand, caffeic and gallic acids were preferentially adsorbed and oxidized from solution regardless the catalyst used, either by the higher chelating activity of Fe towards such compounds, and/or by the preferential electrophilic attack of hydroxyl radicals to the phenol’s aromatic rings. Under the experimental conditions tested, the greater TPh removals were achieved using M-Fe and N-Fe catalysts prepared from commercially available AC supports, reaching 92% for M-Fe. Nonetheless, Fe leaching values in this case was ca. 3.5 mg/L (corresponding to >5 wt.%), which leads to the inevitable contribution of the homogeneous process and catalyst’s deactivation. In the case of N-Fe, the TOC mineralization degree was significantly lower than the TPh removal, which indicates the formation of refractory intermediates. Using Fe-AC catalysts derived from agricultural wastes, the total phenolic content oxidation ranged from 50–56%, but catalysts showed an improved stability and favored the total mineralization of pollutants.

The toxicity of the solutions decreased linearly with TPh reduction. Among the range of materials prepared from organic residues, the physically-activated olive stone catalyst (OSC-AC-Fe) seems to be the most promising, showing a mineralization degree (TOC) and efficiency in the H_2_O_2_ consumption comparable to N-Fe and M-Fe catalysts, also reducing the toxicity of the initial solution towards the *V. fischeri* bacteria by >60%. Supports and Fe-active phases are being modified by different activation treatments and/or functionalization in order to improve specific interactions with OMW pollutants, which demonstrated to control adsorption rate and further oxidation degree.

## Figures and Tables

**Table 1:** Summary of synthesis conditions, nomenclature of catalysts and yield values of the carbonization/activation processes.

Catalyst	Starting Material	Carbonization	Activation Agent	Carb./Act. Yield (%)
OSC-Fe	Olive Stone	800 °C/N_2_/2 h	-	24
OSC-AC-Fe	Olive Stone	800 °C/N_2_/2 h	CO_2_	16
OS-AC/KOH-Fe	Olive Stone	-	KOH	14
SDC-Fe	Sawdust	800 °C/N_2_/2 h	-	23
SDC-AC-Fe	Sawdust	800 °C/N_2_/2 h	CO_2_	16
N-Fe	Norit RX3 Extra	Commercial, used as received		-
M-Fe	Merck	Commercial, used as received		-

**Table 2:** Physicochemical characterization of the synthetic effluent; overview of olive mill wastewater (OMW) characteristics from different sources: storage pond (weathered), olives washing, and olive oil extraction centrifuges.

OMW Source	pH	COD (g/L)	BOD_5_ (g/L)	TOC (g/L)	TPh (g/L)	TSS (g/L)	References
Synthetic solution	3.8	0.77	0.19	0.21	0.35	-	This work
Storage pond (weathered)	6.3	1.7	0.47	0.31	0.18	0.25	[46]
Olives washing	6.3–7.2	0.8–4.1	0.3–1.5	-	0.04–0.10	8–18	[21,57]
Extraction centrifuges	3.5–6.0	4–200	0.8–100	8.3–26.0	0.1–7.4	2–35	[21,47,58,59]

COD—Chemical Oxygen Demand; BOD_5_—Biochemical Oxygen Demand after 5 days; TOC—Total Organic Carbon; TPh—Total Phenolic Content (expressed as caffeic acid equivalents); TSS—Total Suspended Solids.

**Table 3:** pH_pzc_ and textural characteristics of supports and corresponding catalysts.

Sample	pH_pzc_	*S*_BET_ (m^2^/g)	*W*_0_ (N_2_) (cm^3^/g)	*L*_0_ (N_2_) nm	*W*_0_ (CO_2_) (cm^3^/g)	*V*_meso_ (cm^3^/g)	*V_T_* (cm^3^/g)
OSC	10.3	136	0.06	*n.a.*	0.17	0.08	0.17
OSC-Fe	2.2	10	0.01	*n.a.*	0.16	0.03	0.04
OSC-AC	10.6	792	0.33	1.2	0.20	0.04	0.39
OSC-AC-Fe	2.4	546	0.23	1.4	0.13	0.05	0.30
OS-AC/KOH	8.3	1013	0.43	1.7	0.18	0.09	0.55
OS-AC/KOH-Fe	2.0	526	0.23	2.0	0.17	0.08	0.33
SDC	11.5	82	0.04	*n.a.*	0.11	0.08	0.14
SDC-Fe	4.2	9	0.00	*n.a.*	0.10	0.02	0.03
SDC-AC	10.7	565	0.24	1.6	0.15	0.10	0.39
SDC-AC-Fe	3.2	176	0.08	*n.a.*	0.12	0.10	0.21
N	11.6	1058	0.44	1.6	0.21	0.08	0.55
N-Fe	2.4	777	0.33	1.7	0.23	0.07	0.42
M	7.0	831	0.35	1.5	0.25	0.07	0.44
M-Fe	1.9	663	0.28	1.6	0.22	0.06	0.36

*S*_BET_: BET surface area; *W*_0_: micropores’ volume; *L*_0_: mean micropores’ width; *V*_meso_: mesopores’ volume; *V_T_*: total pore volume (*P*/*P*_0_ = 0.95); *n*.*a*.: not applicable.

**Table 4:** Atomic surface composition determined by X-ray photoelectron spectroscopy (XPS) analysis of the prepared catalysts.

Catalyst	Atomic Content (%)				
	C	O	N	Fe	Others
OSC-Fe	91.5	7.2	0.3	0.8	0.2
OSC-AC-Fe	88.5	9.6	0.2	1.6	0.1
OSC-AC-Fe used	75.8	20.8	1.5	1.9	-
OS-AC/KOH-Fe	86.9	10.7	0.4	1.7	0.3
SDC-Fe	80.8	13.7	-	2.4	3.1
SDC-AC-Fe	77.5	15.7	0.7	4.6	1.5
N-Fe	76.2	17.4	0.3	4.2	1.9
M-Fe	89.8	8.0	0.3	1.8	0.1

**Table 5:** Distribution of oxygen and iron species on the catalysts’ surface.

Peak (eV)	OSC-Fe	OSC-AC-Fe	OSC-AC-Fe Used	OS-AC/KOH-Fe	SDC-Fe	SDC-AC-Fe	M-Fe	N-Fe
**O1_s_**	*Area %*							
Fe–O (530.1)	12	20	8	20	37	35	34	30
C=O (531.6)	58	48	33	43	43	44	30	27
C–O (533.2)	30	32	59	37	20	21	36	43
**Fe_2_p_3/2_**	*Area %*							
Fe^2+^ (709.9)	13	22	29	25	29	23	27	20
Fe^3+^ (710.9)	30	45	32	40	28	37	36	41
Fe^3+^ (712.0)	34	19	18	18	28	17	19	21
Fe^3+^ (713.0)	13	10	16	15	9	12	13	11
Fe^3+^ (714.1)	10	4	5	2	6	11	5	7

## Supplementary Materials

The following are available online at https://www.mdpi.com/2079-4991/10/5/876/s1: Table S1: Chemical characteristics of the selected phenolic compounds; Figure S1: N_2_ adsorption/desorption isotherms for the supports and corresponding Fe-catalysts tested; Figure S2: C1s spectral region of OSC-Fe and OSC-AC-Fe catalysts; Figure S3: Adsorption runs using the catalysts prepared: removal of each phenolic compound (C/C_0_) over time. Experimental conditions: [Cat] = 0.5 g/L, T = 25 °C, pH = unadjusted; Figure S4: Catalytic effect of H_2_O_2_ on the removal of phenolic compounds (C/C_0_) over time. Experimental conditions: [H_2_O_2_] = 1 g/L, T = 25 °C, pH = unadjusted; Figure S5: Catalytic runs using the Fe/AC catalysts prepared: removal of phenolic compounds (C/C_0_) over time. Experimental conditions: [H_2_O_2_] = 1 g/L, [Cat] = 0.5 g/L, T = 25 °C, pH = unadjusted.
