# Supplementary material for: Fitting Biochars and Activated Carbons from Residues of the Olive Oil Industry as Supports of Fe- Catalysts for the Heterogeneous Fenton-Like Treatment of Simulated Olive Mill Wastewater
Source: Nanomaterials (Basel). 2020 May 1;10(5):876. doi: 10.3390/nano10050876 (PMC7279504; doi:10.3390/nano10050876)
Supplement: Supplementary file 1 [file nanomaterials-10-00876-s001.pdf]

nanomaterials

Supplementary Information

Fitting biochars and activated carbons from residues of the olive oil industry as supports of Fe-catalysts for the heterogeneous Fenton-like treatment of simulated olive mill wastewater

Bruno M. Esteves [1], Sérgio Morales-Torres [2], Francisco J. Maldonado-Hódar [2] and Luis M. Madeira [2]

[1] LEPABE – Laboratory for Process Engineering, Environment, Biotechnology and Energy, Faculty of Engineering, University of Porto, Rua Dr. Roberto Frias, 4200-465 Porto, Portugal; ega11010@fe.up.pt (B.M.E); mmadeira@fe.up.pt (L.M.M)

[2] Carbon Materials Research Group, Department of Inorganic Chemistry, Faculty of Sciences, University of Granada, Avda. Fuente Nueva, 18071 Granada, Spain; semoto@ugr.es (S.M-T), fjmaldon@ugr.es (F.J.M-H)

Table S1. Chemical characteristics of the selected phenolic compounds.

Name	Chemical structure	Molecular formula	Molecular weight (g/mol)	pKa (at 25 ºC)	Solubility in water (g/L)
Tyrosol	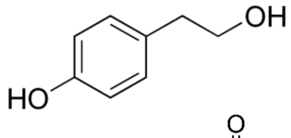	$C_8H_{10}O_2$	138.16	10.20	25.3
Caffeic acid	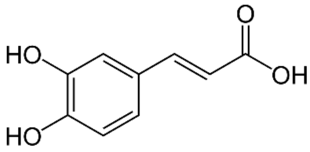	$C_9H_8O_4$	180.16	4.62	1.6
Gallic acid	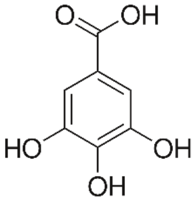	$C_7H_6O_5$	170.12	4.40	11.5
Protocatechuic acid	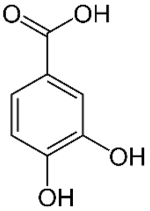	$C_7H_6O_4$	154.12	4.48	18.2
Vanillic acid	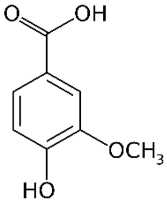	$C_8H_8O_4$	168.14	4.16	1.5

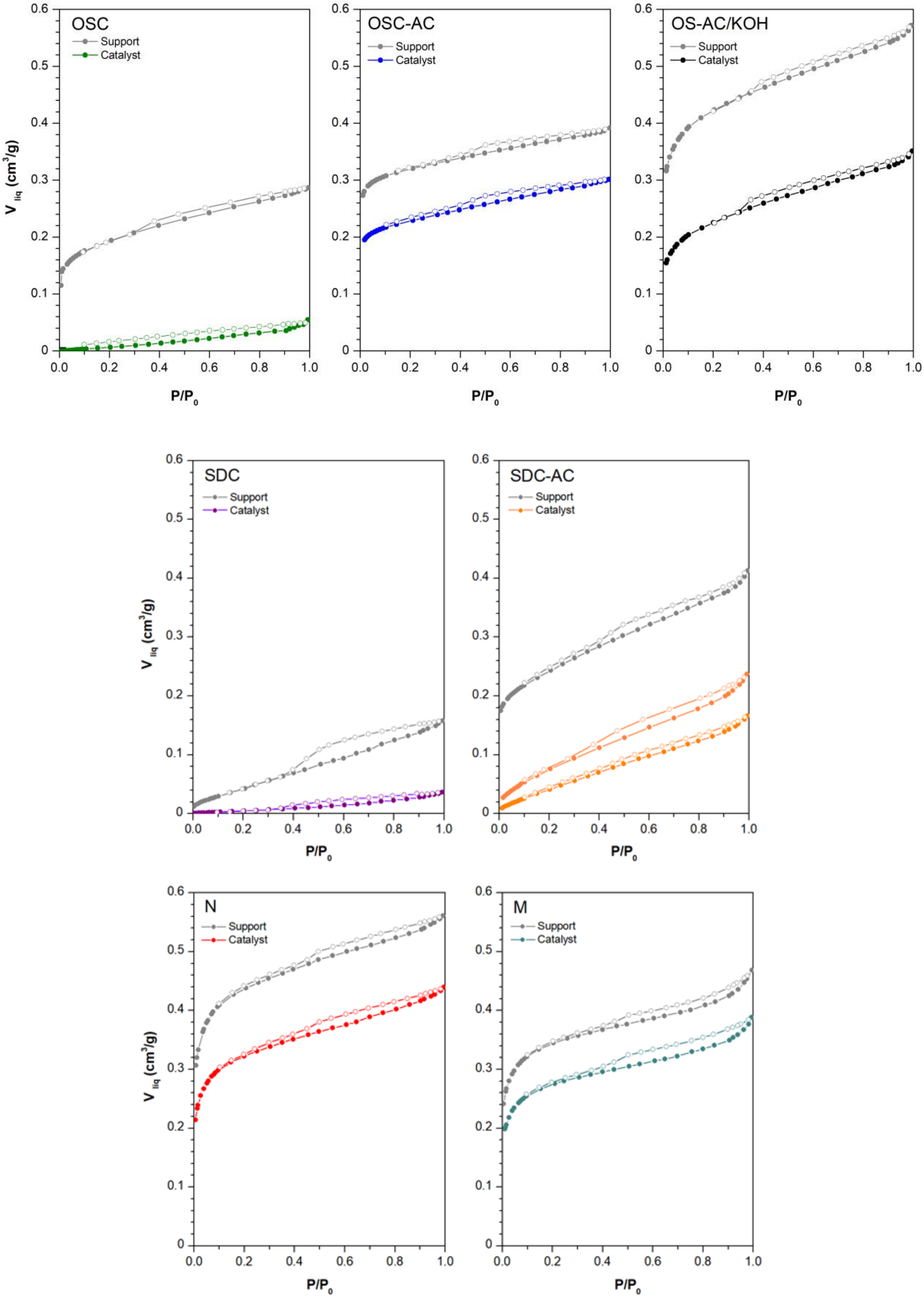

Figure S1. N_2 adsorption/desorption isotherms for the supports and corresponding Fe-catalysts tested.

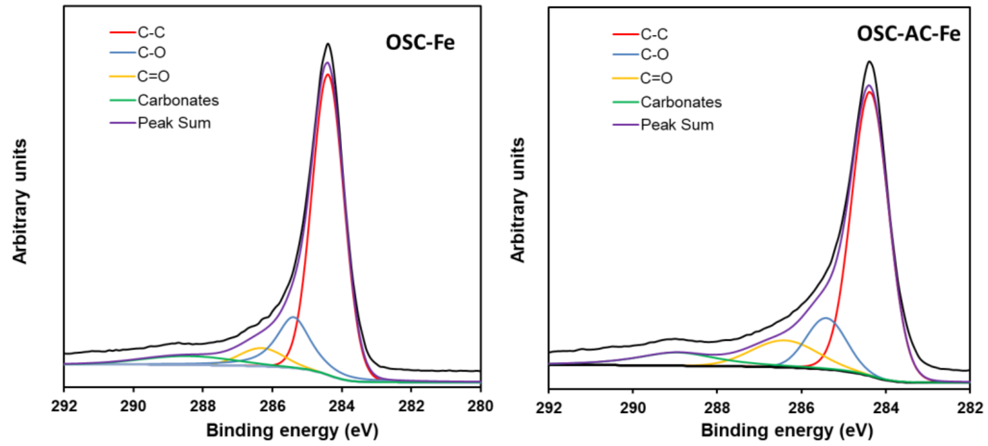

Figure S2. C1s spectral region of OSC-Fe and OSC-AC-Fe catalysts.

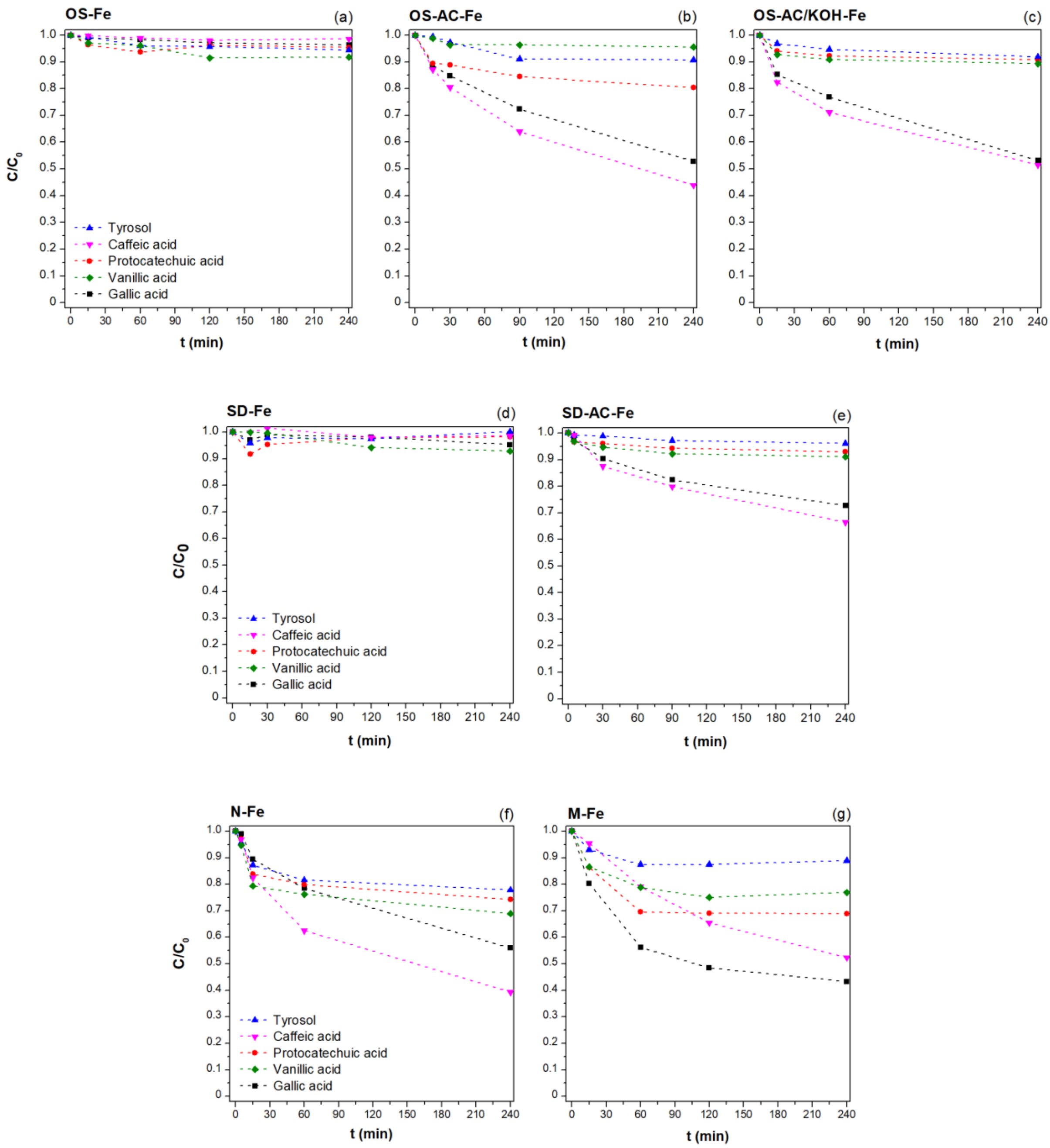

Figure S3. Adsorption runs using the catalysts prepared: removal of each phenolic compound (C/C0) over time. Experimental conditions: [Cat] = 0.5 g/L, T = 25 ºC, pH = unadjusted.

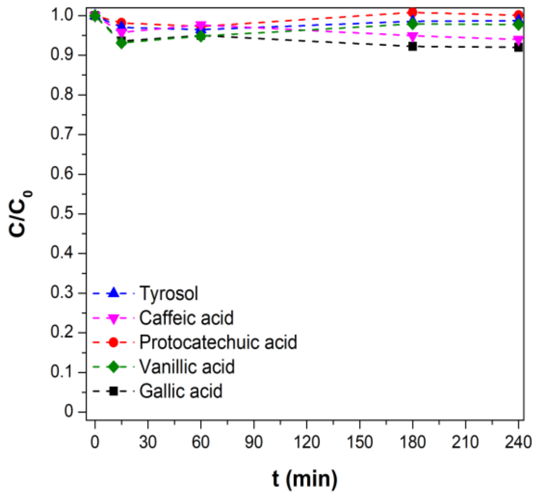

Figure S4. Catalytic effect of H_2O_2 on the removal of phenolic compounds (C/C_0) over time. Experimental conditions: [H_2O_2] = 1 g/L, T = 25 ^{0}C, pH = unadjusted.

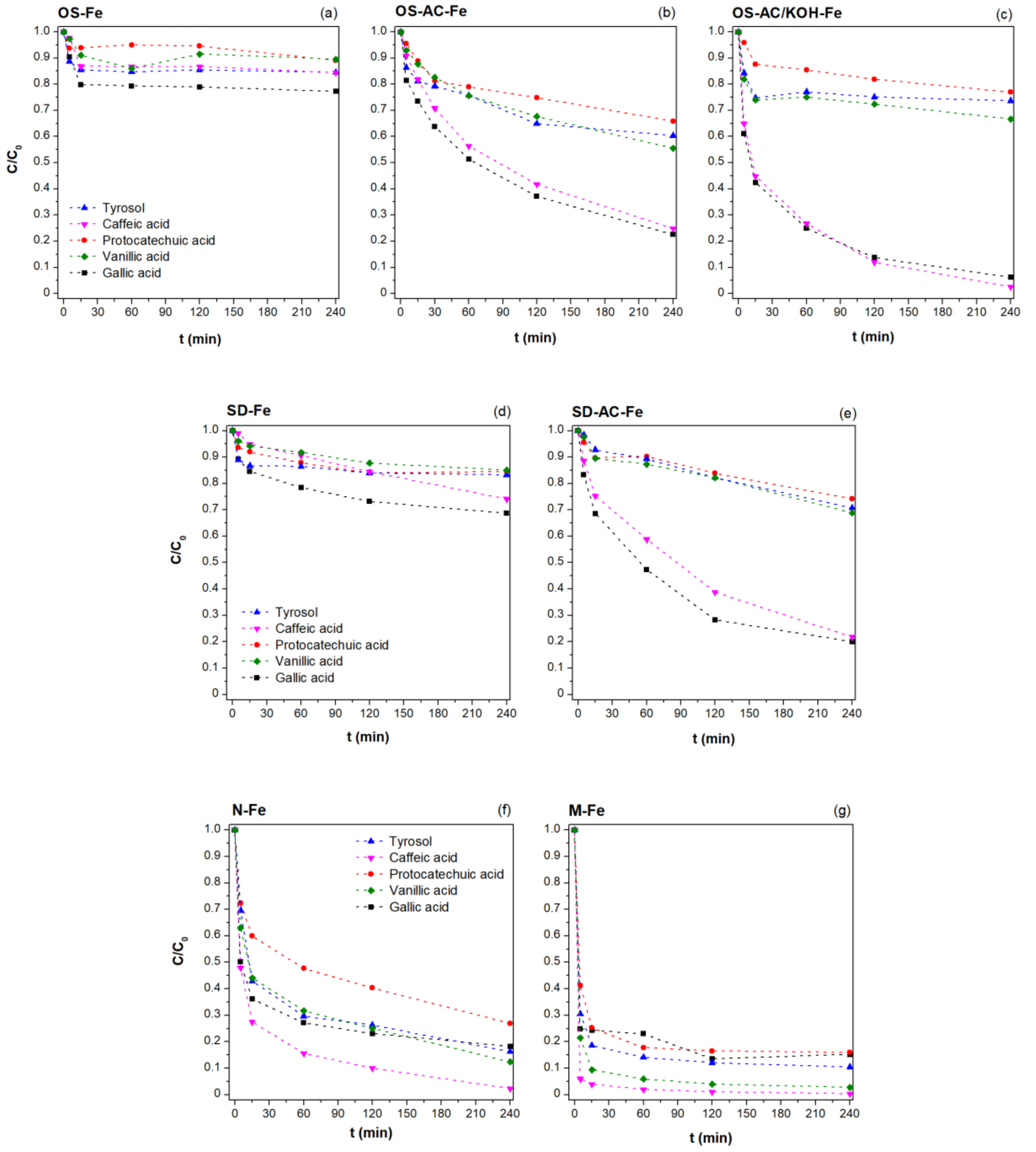

Figure S5. Catalytic runs using the Fe/AC catalysts prepared: removal of phenolic compounds (C/C_0) over time. Experimental conditions: $[H_2O_2]$ = 1 g/L, [Cat] = 0.5 g/L, T = 25 ^{0}C, pH = unadjusted.
